# Engineering protein-based therapeutics through structural and chemical design

**DOI:** 10.1038/s41467-023-38039-x

**Published:** 2023-04-27

**Authors:** Sasha B. Ebrahimi, Devleena Samanta

**Affiliations:** 1 Drug Product Development—Steriles, GlaxoSmithKline, Collegeville, PA 19426 USA; 2 Department of Chemistry, The University of Texas at Austin, Austin, TX 78712 USA

**Keywords:** Drug delivery, Protein design, Proteins

## Abstract

Protein-based therapeutics have led to new paradigms in disease treatment. Projected to be half of the top ten selling drugs in 2023, proteins have emerged as rivaling and, in some cases, superior alternatives to historically used small molecule-based medicines. This review chronicles both well-established and emerging design strategies that have enabled this paradigm shift by transforming protein-based structures that are often prone to denaturation, degradation, and aggregation in vitro and in vivo into highly effective therapeutics. In particular, we discuss strategies for creating structures with increased affinity and targetability, enhanced in vivo stability and pharmacokinetics, improved cell permeability, and reduced amounts of undesired immunogenicity.

## Abstract (web summary)

Ebrahimi and Samanta review the key advances in the chemical and structural modification of proteins that have enabled their rise as indispensable tools in medicine and outline emerging protein engineering strategies that can potentially unlock structures with improved therapeutic properties.

## Introduction

In 1982, Humulin became the first FDA-approved, recombinant, protein-based therapeutic^1^. Today, protein-based drugs constitute a market approaching ~$400 billion with hundreds of candidates approved and in clinical trials^2^. Here, we chronicle the chemical design strategies that have transformed the field of medicine from one that has been historically dominated by small molecule pharmaceuticals to one where proteins have emerged as comparable or superior rivals.

Proteins represent the most versatile class of biomolecules, acting as catalysts, signaling molecules, molecular and ion transporters, scaffolds for maintaining cellular and tissue integrity, receptors, and more^3^. Therefore, as medicines, proteins can be used to serve any of these roles (Box 1) and offer several advantages over small molecule drugs. Proteins are less likely to cause side effects by interfering with normal biological processes because they have evolved to play highly specific roles. Typically, protein therapeutics show high potency and can also execute more complex functions owing to their intricate three-dimensional structures.

**Fig. 1:**
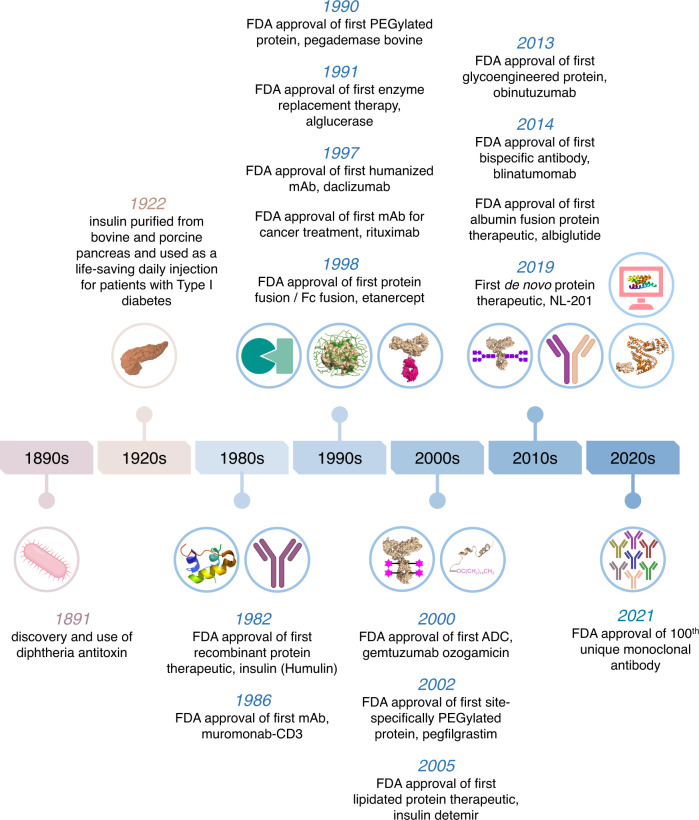
A timeline of significant advances in the development of protein-based therapeutics.

Over the last ~150 years, fundamental advances have been made towards harnessing the power of proteins to create new medicines (Fig. 1). The development of an antibody-based treatment for diphtheria in 1891 was a major milestone and was awarded the first Nobel Prize in medicine^4^. The antibody was extracted from the serum of horses that had been challenged with an attenuated form of diphtheria-causing bacteria. Two decades later, the extraction of insulin from porcine pancreas for the treatment of diabetes mellitus marked another significant advance—the use of an exogenous protein to treat an endogenous deficiency^5,6^. However, two tons of pig pancreas were required to produce eight ounces of protein^7^. Assuming 72 million patients (a close approximation for the number of diabetics in the world requiring insulin) and the need for 0.02 ounces of insulin per year for the average patient, this would necessitate ~150 million pigs/yr, making it challenging to produce the drug at appropriate scale^8,9^. Moreover, the use of proteins extracted from animals could elicit an immune response in patients and lead to potential exposure to animal diseases. A breakthrough moment came in 1982 when recombinant DNA technology was used to produce insulin in a bacterial host (*E. Coli*)^10^. The successful use of recombinant DNA technology helped to circumvent challenges with both scale-up and immunogenicity of animal-derived proteins. Considering that, in principle, one could express any protein for which its associated gene is known, a viable competitor to the workhorse of medicine at the time – small molecules – had truly emerged.

Still, there were intrinsic challenges that needed to be solved due to the inherent susceptibility of proteins to aggregation, degradation, denaturation, and concomitant loss of activity^11^. Moreover, untimely clearance from the body, non-specific distribution, immunogenicity, and toxicity also posed relevant concerns^12^. Advances in rational design and ability to deliberately introduce chemical and structural modifications have driven a paradigm shift in how these properties can be tuned^13–15^. In the following sections, we delineate the key considerations that drive the chemical design of protein-based therapeutics and describe the various design strategies that have led to ~350 approved drug products (Supplementary Tables S1–S3). We end with an outlook on areas that need further development to realize the full potential of these molecules. We particularly focus on deliberate structural and chemical modifications that are made directly to the protein structure as opposed to strategies such as directed evolution and protein encapsulation, or those focusing on tuning protein formulations. We also exclude vaccines.

Box 1 Common classes of protein-based therapeutics **Antibodies**

Antibodies are produced by B lymphocyte cells of the immune system in response to foreign objects, such as invading pathogens. They function by binding to specific molecules on the pathogen’s surface (antigens) and inactivating the invader. As therapeutics, antibodies are predominantly used to bind target antigens which can then block specific signaling pathways (e.g. nivolmab) or induce cell death (e.g. binutuzumab). Antibodies can also serve as vehicles for the targeted delivery of potent cytotoxic drugs to diseased cells (e.g. antibody-drug conjugates such as trastuzumab emtansine). Monoclonal antibodies (mAbs), generated using clones of a unique lymphocyte cell, constitute the most prevalent type of antibody-based therapeutics. Structurally, antibodies are Y-shaped, comprising two antigen-binding fragments (Fab) and a crystallizable fragment (Fc).

**Enzymes**

Enzymes play a key role in the human body by catalyzing a wide variety of biochemical reactions. As therapeutics, they can be delivered to replace a deficient or absent enzyme in so called enzyme replacement therapy. In other cases, they can be used as agents to catalyze the degradation, cleavage, or chemical modification of therapeutically relevant targets. Examples of enzyme-based drugs include PEG-asparaginase, sacrosidase, pegvaliase, and laronidase.

**Coagulation factors**

Coagulation factors are naturally occurring proteins that play an essential role in the blood clotting process. Consequently, as therapeutics, these proteins can be plasma-derived or produced recombinantly and administered to patients with a deficiency in native levels. Coagulation factor-based medicines are used for various bleeding-related indications, including hemophilia A and B (e.g. eptacog alfa).

**Protein hormones**

Protein hormones constitute a class of molecules that are secreted by endocrine glands. They act as chemical messengers by binding to receptors which then triggers a signaling cascade and leads to a physiological response. Insulin represents a classic example. After a meal, insulin facilitates glucose removal from the bloodstream by binding to its cell-surface receptor. This initiates an intracellular cascade that results in translocation of glucose transporters to the surface and subsequent uptake of glucose into cells. Other therapeutically-relevant protein hormones include erythropoietin and gonadotropin.

**Cytokines**

Cytokines comprise a broad group of proteins (encompassing molecules such as interleukins, interferons, and colony-stimulating factors) that mediate cell-to-cell communication during immune responses. This contrasts with protein hormones, which largely regulate the endocrine system (vide supra). Therapeutic cytokines can be used as immunomodulatory agents for a variety of indications, including multiple sclerosis (interferon β−1b) and hairy cell leukemia (interferon α−2b).

### Key considerations driving the chemical design of protein-based therapeutics

**Fig. 2:**
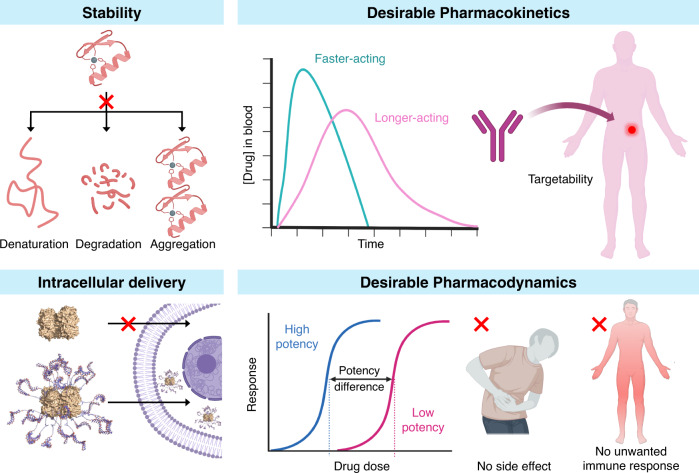
Desirable characteristics of protein-based therapeutics. These include high stability (top left), ability to enter cells (bottom left), and appropriate pharmacokinetics (top right) and pharmacodynamics (bottom right).

To function as effective therapeutics, proteins must have certain characteristics (Fig. 2). These desirable attributes vary from protein to protein based on the application of interest. A key consideration is the stability of the proteins both under storage conditions and in vivo. Many proteins are susceptible to aggregation, degradation (e.g., via deamidation/oxidation in vitro, through proteases in vivo, etc.), and denaturation which can significantly reduce efficacy^16,17^. Some proteins are sensitive to moderate changes in temperature, which is an added concern for their transport and storage in different locations^11^. Moreover, the residues at the surface of certain proteins can display favorable interactions with container surfaces, resulting in adsorption and reducing the concentration of the active ingredient available for therapeutic action^18^. Therefore, several structural modifications (e.g., site-specific mutations^19^ and PEGylation^20^) are aimed at improving the solubility and stability of proteins.

Protein-based therapeutics must also exhibit appropriate pharmacokinetics and pharmacodynamics for optimal function. For example, a protein that is rapidly cleared from the body is not suitable for applications that require sustained action^16^. On the other hand, a protein that circulates for a very long time may cause side effects. Similarly, protein drugs must not interfere with the body’s natural functions or elicit unwanted immune responses. To address these challenges, various strategies including PEGylation^20^, glycosylation^21^, lipidation^22^, and protein fusion^3^ have been developed.

Another important consideration in the development of protein-based medicines is targetability. The ability to target drugs to specific tissues (e.g., cancer cells) or organs (e.g., brain) is highly desirable both from the standpoint of lowering therapeutic dose as well as reducing side effects. For example, antibody-drug conjugates can be used to direct the drug to cells that express a specific receptor as the antigen. Many protein-based structures are prone to sequestration in the liver, kidney, and spleen^23^. Strategies wherein targeting moieties are incorporated to promote distribution outside of these organs are an active area of investigation^24^. Recently, it has been shown that proteins covalently conjugated to multiple copies of the transferrin aptamer show preferential accumulation in the brain relative to native proteins^25^.

The majority of current protein-based pharmaceuticals have extracellular targets. The relatively large sizes compared to small-molecule drugs and heterogeneous surface charges render most proteins impermeable to the cell-membrane. Intracellular delivery is highly sought after as the ability to intercept deleterious intracellular processes can lead to highly effective medicines. Emerging chemical strategies such as appending cell-penetrating peptides^26^, supercharging^27^, and dense DNA grafting^28^ have shown promising results in this regard.

**Fig. 3:**
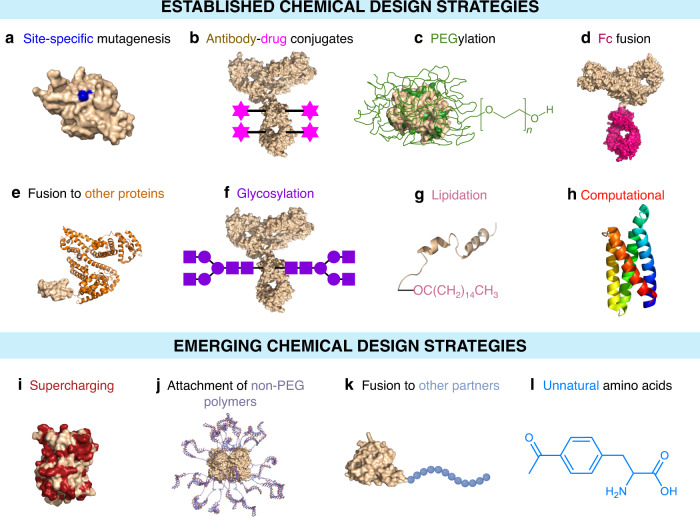
Established and emerging chemical design strategies employed to generate protein-based therapeutics. **a** Site-specific mutagenesis. **b** Antibody-drug conjugates. **c** PEGylation. **d** Fc fusion. **e** Fusion to other proteins. **f** Glycosylation. **g** Lipidation. **h** Computational. **i** Supercharging. **j** Attachment of non-PEG polymers. **k** Fusion to other partners. **l** Unnatural amino acids. The structure shown in (**h**) represents neoleukin (NL)−2/15 (PDB ID: 6DG6) which is a predecessor to NL-201, the world’s first de novo protein therapeutic.

While chemical modifications to protein structures can impart several advantageous properties, they can also alter the activity or potency of the drugs^20^. Typically, high activity or potency is a desirable trait as it can lower the therapeutic dose necessary. The central challenge in designing protein-based therapeutics is to choose a strategy that leads to the gain of a certain function without causing the loss of another. Table 1 summarizes the ability of established and emerging chemical design strategies (Fig. 3) to achieve this balance.

**Table 1:**
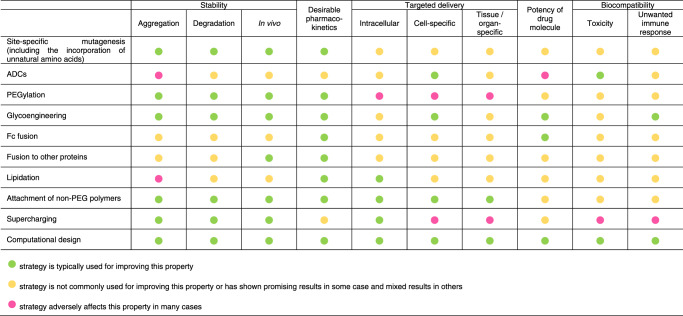
Structural and chemical design strategies and their impact on the properties of protein-based therapeutics

### Established chemical design strategies

#### Site-specific mutagenesis

The use of site-specific mutagenesis to introduce amino acid point mutations has been a widely employed method to confer enhanced properties to protein-based therapeutics^29^. A classic example of this has been in the development of insulin variants with different kinetics of action. (Supplementary Table S1). For instance, the substitution of asparagine by glycine at amino acid 21 of the α chain and the addition of 2 arginines to the β chain gives rise to insulin glargine, a long-acting variant with duration of action up to 24 h^30^. These amino acid modifications increase the isolectric point (pI) of the structure towards physiological pH, resulting in precipitation upon injection and therefore a decrease in absorption rate^29^. In other cases, substitutions can be made that decrease self-association and increase the rate of absorption^30^. Insulin glulisine, for example, has a modified amino acid sequence wherein β chain asparagine (position 3) and lysine (position 29) are exchanged with lysine and glutamic acid, respectively^31^. The subsequent decrease in pI from 5.5 (native insulin) to 5.1 promotes increased solubility with less propensity for hexamer formation, leading to fast-acting effect.

Targeted mutations can also be used for tuning protein stability in solution, resulting in more robust formulations less prone to unwanted aggregation. A well-established modification involves the substitution of free cysteines with other amino acids such as serine to prevent the formation of non-native disulfide bonds or oxidation of cysteine residues leading to thiol adducts or cysteic acid^32^. This strategy has been employed in various FDA-approved protein therapeutics, including aldesleukin (Proleukin), interferon β1b (Betaseron), and pegfilgrastim (Neulasta). To explore a more general strategy, Trout et al. developed spatial aggregation propensity (SAP), a molecular dynamics-based simulation technique for identifying key regions in proteins that drive aggregation^33^. Based on this information, they were able to make specific mutations that enhanced stability compared to the native protein.

Validated point mutations are also commonly used to control IgG-based antibody behavior in vivo. For instance, circulation half-life can be tuned by introducing substitutions into the Fc region that change the nature of binding interactions with the neonatal Fc receptor (FcRn), a receptor present in the endosomes of a variety of cell types. Specifically, promoting binding at endosomal pH (<~6.5) leads to the antibody being trafficked to the cell surface with the FcRn rather than being sent to the lysosome for degradation. At the cell surface, the increase in pH results in loss of binding affinity and release of the antibody into circulation. Taken together, this “recycling” mechanism mediated by FcRn plays a key role in dictating the circulation half-life of antibodies. By tuning the binding strength of Fc to FcRn, circulation half-life can be increased or decreased^34^. Fc domains with the amino acid substitutions M428L/N434S (LS variant) and M252Y/S254T/T256E (YTE variant) constitute two common examples for such modifications^34,35^. Notably, the LS variant has been used in the FDA-approved ravulizumab (Ultomiris) to increase circulation half-life in comparison to the parent antibody, eculizumab (Soliris)^36^. Furthermore, the discovery of new advantageous mutations remains an active area of research. Beyond tuning half-life, Fc mutations can also be made for either increasing or decreasing antibody effector function (e.g. antibody-dependent cellular cytotoxicity (ADCC), antibody-dependent cellular phagocytosis (ADCP), complement-dependent cytotoxicity (CDC)). A notable example is the fusion protein abatacept (Orencia) for arthritis, where C220S/C226S/C229S/P238S mutations in the linked Fc moiety led to significant decrease of cytotoxicity associated with CDC and ADCC^37^.

The main challenge with introducing site-specific mutations is that the mutation may negatively impact structure (i.e. prevent proper protein folding, decrease conformational/colloidal stability) or lead to decreased protein function. Furthermore, in some cases, it can be difficult to predict a priori how mutations might affect protein stability and function.

#### Antibody-drug conjugates (ADCs)

ADCs constitute an emerging class of medicines that are generally used in the treatment of various forms of cancer. These structures consist of an antibody conjugated to a cytotoxic drug via a linker module. Attachment of the cytotoxic drug to the antibody is commonly achieved via modification of reactive residues such as cysteines or lysines. ADCs use the antibody component as the targeting moiety (e.g., to bind an antigen present in high abundance on cancer cells) to achieve cell-selective delivery of potent drug payloads that would be too toxic for administration on their own. This method increases the payload’s therapeutic index^38^. The linker plays the critical role of keeping the cytotoxic payload conjugated to the antibody while in circulation as premature release could result in significant toxicity^38^. Once at the target site (e.g. within a cancer cell), the linker should release the payload to allow for therapeutic effect to take place. While trials in patients began in the 1980s, challenges such as in vivo immunogenicity, in vitro instability, and lack of sufficient potency historically made ADC translation into the clinic difficult^15^. Continuous advances in knowledge in these challenging areas have resulted in the approval of 12 ADCs over the last ~11 years (Supplementary Table 2)^38^. A particularly interesting example is Kadcyla, approved by the FDA in 2013. Kadcyla consists of the FDA-approved trastuzumab (Herceptin) conjugated to the chemotherapeutic emtansine, illustrating the utility of repurposing a clinically validated antibody as the targeting element in the design of ADCs. Moreover, early efforts in ADC development utilized drug payloads that substantially lost potency upon conjugation to antibodies. This largely arose from differences in cellular uptake mechanisms between free and attached payloads. Specifically, while a hydrophobic drug molecule on its own can diffuse into cells in large quantities, its conjugation to an antibody limits its uptake while also requiring an additional release step from the antibody in order to initiate a therapeutic effect. The use of even more potent payloads, such as auristatin-based drugs, helped overcome these challenges. Other advances, including the design of linkers that effectively release payloads intracellularly, were also pivotal in the rise of ADC therapeutics^38,39^.

Several areas of research remain open in the ADC field. Conjugation of payloads to lysine or cysteine residues results in structures with varying drug loadings, meaning that a mixture of species is administered to patients^40,41^. This is especially important to consider as each drug-loading variant will have a unique profile with regards to toxicity, aggregation propensity, pharmacokinetics, target affinity, and potency^40^. Consequently, the exploration of strategies for site-specific conjugation of antibodies to achieve uniform drug loading remains a continuous area of work. Beyond several ADCs being tested in clinical trials, the development of new ADCs for various indications such as neuroblastoma^42^, colon cancer^43^, and hepatocellular carcinoma^44^ remains ongoing.

#### PEGylation

One of the most common strategies for extending the half-life of protein therapeutics is the attachment of polyethylene glycol chains (PEGylation) to the surface. PEGylation increases the overall hydrodynamic diameter. As kidneys filter molecules based on size (e.g., particles with hydrodynamic diameters below 5–6 nm are rapidly cleared), PEGylation decreases the clearance rate of the drug from the body. Moreover, PEGylation can enhance solubility, protect against proteolytic degradation or shield immunogenic epitopes from recognition by the immune system^20^. The first PEGylated protein, adenosine deaminase (Adagen), was approved in 1990 with several more cleared by the FDA over the course of the next three decades^3^.

In some cases, previously approved proteins have been PEGylated to create longer acting variants that require less frequent administration. For example, turoctocog alfa (NovoEight, FDA-approved in 2013), used for treating hemophilia A, was transformed into a longer-acting drug turoctocog alfa pegol (Esperoct, FDA approval in 2019)^45^. Early work in the PEGylation field largely modified proteins in a nonspecific fashion through conjugation of amino handles on lysines and the N-terminus^20^. Moreover, the PEG starting materials generally consist of a mixture of various length chains. This presents a challenge as the resultant heterogeneous pool of products can have vastly differing therapeutic properties. In this regard, later efforts focused on the site-specific modification of proteins towards overcoming these drawbacks^46^. One example is certolizumab pegol (Cimzia), containing an engineered unpaired cysteine for conjugation to PEG^47^.

PEGylation can negatively affect protein activity, in some cases with loss of up to ~99% activity^48^. Moreover, some reports have claimed that PEG itself can be immunogenic, which has motivated research into the use of non-PEG polymers as an alternative (*vide infra*)^49^. PEGylation also raises concerns about unwanted PEG accumulation in vivo. To alleviate this issue, some researchers have explored the incorporation of biocleavable moieties into PEG chains^14,50^.

#### Fc fusion

Fc-fusion proteins constitute a class of engineered therapeutics wherein the Fc region of an antibody is fused to a biologically active protein or peptide. Typically, Fc-fusion is done to enhance circulation half-life. The Fc moiety is able to extend circulation half-life by increasing the hydrodynamic diameter of the fusion-protein to slow kidney filtration and via interaction with the FcRn receptor. Two examples of this approach are efmoroctocog alfa (Eloctate) and eftrenonacog alfa (Alprolix), both of which are FDA-approved. Compared to conventional coagulation Factor VIII proteins, Eloctate exhibits ~1.4–1.8 fold enhanced circulation time, leading to a less frequent requirement for patient injection^51^. Alprolix shows a more significant change, exhibiting ~3-fold higher circulation time than unmodified Factor IX protein^52^. The addition of the Fc moiety can also help drive effector response (ADCC, ADCP, etc.) towards the creation of more potent protein therapeutics^53,54^. Another potential benefit of Fc fusions is illustrated by etanercept (Enbrel), an FDA-approved TNF-α blocker for treating inflammatory conditions. Here, the TNF-α receptor is fused to an Fc moiety which then dimerizes due to disulfide bond formation between Fc groups. This dimerization leads to a structure that has significantly higher affinity for TNF-α compared to the receptor alone^55^.

Fc fusions offer the benefit of being able to genetically encode the entire structure. This is in contrast to PEGylation, where PEG moieties are chemically conjugated after protein expression. Drawbacks include the fact that incorporation of an Fc component can elicit effector response (ADCC, ADCP, etc.) even in situations where this is not desirable^56^. The Fc region can also be prone to degradation by proteases in vivo.

#### Fusion to other proteins

A common strategy for increasing the half-life of a protein therapeutic involves fusing it to another protein with a long circulation time. Human serum albumin (HSA) has been a widely used fusion partner owing to its relatively long half-life of ~19 days^57^. Moreover, conjugation to HSA increases the size of the structure reducing the rate of kidney filtration and protects its fusion partner from potential in vivo protease degradation. Taken together, HSA-fusions confer a significant improvement in the pharmacokinetic profile of a drug. A notable example of a clinical success involves albutrepenonacog alfa (Idelvion), indicated for the treatment of hemophilia B. In this case, the fusion of HSA extends the half-life from ~22 to ~102 h^58^.

Fusing proteins can also lead to the creation of bispecific agents. The FDA-approved T-cell engager, blinatumomab, constitutes one such example. Here, two different single-chain variable fragments (scFv) of an antibody are linked together by a peptide moiety. One scFv recognizes CD3 on T cells, while the other scFv recognizes CD19 on malignant B cells. Therefore, simultaneous binding to both targets bridges cancer cells with activated immune cells. The T cells can secrete various enzymes, leading to cancer cell death^59,60^.

In a similar but distinct example, the FDA-approved caplacizumab constitutes a case of genetically fusing two identical nanobodies via a short amino acid chain to create a bivalent therapeutic agent^61,62^. Nanobodies initially garnered interest after the discovery of antibodies in camelids that contain only heavy chains. These antibodies have a single variable domain that binds antigens. This domain, termed a nanobody, still recognizes its targets when isolated from the whole structure. With a size of around 15 kDa, these small therapeutic agents can potentially access hard to reach areas in vivo, exhibit high stability, and can be produced with relative ease compared to conventional antibodies. A general risk associated with fusion proteins is that the therapeutic protein may lose activity if the binding/active site is occluded upon conjugation^52^.

#### Glycoengineering

Glycosylation is a post-translational modification mediated by intracellular enzymatic machinery wherein oligosaccharide groups are covalently conjugated to the protein structure, most often at asparagine (N-linked) or serine/threonine (O-linked) residues. Certain therapeutic proteins, such as insulin, are not glycosylated and can therefore be expressed in prokaryotic hosts^63^. However, the majority of approved structures are glycosylated. Hosts incapable of glycosylation are not suitable for their expression. In this regard, a key advance in the rise of protein therapeutics was the use of recombinant DNA technology for glycoprotein expression in appropriate host cells. It was found that “appropriate” host cells are typically mammalian – the resultant glycosylated structures were less immunogenic in humans compared to those expressed in other eukaryotic hosts^64^.

Beyond influencing immunogenicity, glycosylation can also impact protein stability, in vivo activity, and pharmacokinetics. The use of glycoengineering has therefore been a well-studied strategy for tuning protein properties^65^. In some cases, glycoengineering involves changing or adding glycosylation sites. One of the most well- known examples is the erythropoietin analog darbepoetin alfa (Aranesp), engineered with two extra locations for N-linked glycosylation^66^. This modification improved the half-life of the drug by ~3-fold owing to an increase in the protein’s size, making kidney filtration slower.

Glycoengineering can also involve changing the identity of the conjugated oligosaccharide groups. In darbepoetin alfa, enhanced half-life is also attributed to its increased sialic acid content, making clearance mediated by asialoglycoprotein receptors (found in liver cells) less favorable^66^. Glycoengineering of antibodies has also yielded several examples of structures with enhanced properties. For instance, Chinese Hamster Ovary (CHO) host cells can be programmed to express antibodies that lack fucose sugar groups (i.e. afucosylation). Afucosylation in the Fc region of antibodies can increase binding to FcγRIIIa receptors. This enhances recruitment and activation of immune effector cells, leading to enhanced ADCC or ADCP potency towards cancer cells. Mogamulizumab (Poteligeo) constitutes one example used in the clinic^66^. While a powerful approach, challenges to optimizing proteins through glycoengineering remain, including difficulty in predicting a priori how changing glycopatterns will influence protein properties and loss of activity upon varying glycosylation identity and sites.

#### Lipidation

The conjugation of lipid groups to proteins has been utilized to enhance therapeutic properties in several approved drugs. Lipidation is often done to enhance pharmacokinetics owing to the ability of lipids to bind to HSA, thereby conferring enhanced characteristics to the structure as a whole. This technique is an alternative to direct fusion of a protein partner to a therapeutic (*vide supra*). A potential advantage offered by this strategy is that the interaction between lipids and HSA is reversible; therefore, the therapeutic protein component of the structure can elicit its effect once no longer bound and sterically blocked by the partner. In certain situations, the presence of the lipid group can also promote reversible multimer formation upon subcutaneous injection that leads to extended release in the body^22^. Insulin detemir was the first lipidated protein to gain FDA approval. It consists of desB30 (i.e. an insulin analog wherein amino acid 30, threonine, is not present) modified with myristic acid at LysB29^67^. Lipidation promotes the formation of dihexamers upon injection that slows absorption and also increases albumin binding, leading to a half-life of ~4–7 h^68^. A more recent and dramatic example of half-life increase is represented by insulin icodec, a lipidated insulin analog investigated in clinical trials that only requires once-a-week administration^69^. Another benefit of lipidation involves its ability to promote intracellular delivery of proteins, which can potentially allow for targeting molecules inside of cells^22^. As is the case with several other modifications, one limitation of lipidation is the potential for loss of protein activity or binding strength if conjugation is close to the active/binding site. Depending on their nature, some lipids may also promote aggregation in vitro or elicit an immune response in vivo^22,70^.

#### Computational design

Protein function can be augmented via computational design^71^. The central assumption driving computational protein design is Anfinsen’s thermodynamic hypothesis—proteins fold into their minimum-energy conformation^72^. Therefore, the effect of varying amino acid sequences on structure can be systematically studied and correlated to function. Computational design can be used to optimize various chemical, physical, and pharmacological properties of therapeutic proteins^73–76^. Specifically, computational design has been shown to improve stability, binding affinity, antibody effector activity, and immunogenicity, among others.

Recently, de novo protein design has emerged as an especially attractive route for designing therapeutic proteins^77^. This allows proteins to be designed from scratch using the fundamental principles of protein biophysics. Whereas the majority of protein engineering focuses on enhancing the functions of existing proteins, often using targeted mutations, de novo protein design focuses on proteins with amino acid sequences not found in nature. The rationale behind this strategy is as follows. A typical protein formed from 200 amino acids can have 20^200^ (≈10^260^) different primary sequences. However, the total number of proteins found in existing organisms is on the order of 10^12^. Therefore, a large portion of sequence space remains unexplored by evolution, and it is reasonable to imagine that sequences within this space may fold to form proteins with novel properties. Efforts in this area have led to the identification of potent mimics of cytokines interleukin-2 (IL-2) and IL-15^78^, programmed cell death protein-1 (PD-1) agonists^79^, and SARS-CoV-2 inhibitors^80^, among others^81,82^. A notable example is the IL-2 mimic NL-201, the world’s first protein therapeutic designed de novo which has shown promise as an anti-cancer immunotherapeutic. Native IL-2 has limited clinical application due to its toxicity which stems from binding to the alpha chain of the IL-2 receptor. In contrast, NL-201 binds exclusively to the beta and gamma chains of the IL-2 receptor which induces the proliferation of anti-tumor effector T cells while avoiding toxicity^78^. In addition, due to structural similarities between the IL-2 and IL-15 receptors, NL-201 is also able to bind to the beta and gamma chains of the IL-15 receptor which causes the expansion of anti-tumor natural killer cells^75–79^.

The main challenge in computational protein design is navigating through the complex conformational energy landscape of proteins (that may contain many local minima) and locating desirable low-energy structures. The method also requires efficient sampling methods, computational power, and continual improvements in machine learning algorithms to accurately predict desired structures.

### Emerging chemical design strategies

#### Supercharged proteins

Supercharged proteins constitute a group of structures containing greater than one net charge for every kilodalton of weight^83^. These highly charged proteins can either be engineered or found in nature and offer unique properties. For example, they show strong resistance to aggregation arising from thermal or chemical stress^27^. This means that upon unfolding due to stresses, supercharged variants can refold and exhibit significant activity even in cases where the unmodified protein cannot^27^. Additionally, positively supercharged proteins can be taken up into cells in large amounts via binding to cell-surface proteoglycans, making them useful for applications where intracellular delivery is desired^84^. Several proteins have been engineered via mutagenesis to create supercharged variants, including glutathione S-transferase and green fluorescent protein (GFP)^83^. Importantly, supercharged proteins can be used as fusion partners to deliver other proteins intracellularly^83^. Moreover, supercharged proteins can be used as fusion partners for functional protein delivery in vivo, including in retinal and pancreatic tissue^27,85^.

Importantly, the residue used for attachment should be chosen such that the supercharged protein does not interfere with the binding or activity of the protein. Furthermore, it should be considered that the use of positively supercharged proteins may lead to toxicity or elicit an in vivo immune response^86^.

#### Attachment of non-PEG polymers

Covalent modification of proteins with polymers other than PEG remains an active area of research. DNA represents a promising option in this regard, wherein dense functionalization of proteins with DNA results in structures called protein spherical nucleic acids (ProSNAs) with several advantageous properties^28^. Firstly, the DNA shell can impart enhanced stability to the structure, both in the form of increased resistance to protease degradation and increased solubility^23^. Secondly, ProSNAs show enhanced circulation times and greater distribution to areas outside of the liver in comparison to the unmodified protein^87^. Finally, the dense arrangement of DNA around the protein core results in recognition by cell surface scavenger receptors, leading to robust cellular uptake up to 280-fold greater than the unmodified protein^23,28,87^. Several reports have utilized ProSNAs, including for the in vivo delivery of highly active proteins and for the intracellular detection of cancer biomarkers^87,88^. One drawback of these structures is that their uptake occurs via endocytosis, meaning that a sufficient quantity must escape the endosome if therapeutic targets are in the cytosol^89^.

#### Other fusion partners

The use of polypeptide chains as fusion partners for protein therapeutics is an active area of research^90–92^. Compared to PEGylated structures, these constructs offer several potential advantages, including the ability to genetically encode their production to obviate the need for chemical conjugation, achieve more homogenous end products, and offer a large design space wherein peptide length and identity can be precisely tuned to produce desirable overall properties. Stemmer et al. reported an early example where they used *E. coli* to express and screen a large library of different polypeptide sequences each containing 864 amino acids^90^. Candidates were assessed for factors such as genetic stability, aggregation propensity, heat sensitivity, and solubility. Based on these criteria, a candidate polypeptide termed XTEN was used as a fusion partner and its potential to impart advantageous properties was assessed. The XTEN moiety was shown to be non-immunogenic even in cases where PEGylation elicited an immune response, yielded a ~ 12-fold increase in circulation time when fused to GFP, and had robust solubility that conferred enhanced stability to its payload partner. Notably, the half-life was highly dependent on the length of the XTEN sequence, offering a tunable way to change pharmacokinetic properties. Efanesoctocog Alfa, a protein-based therapeutic employing the XTEN technology, was recently given breakthrough therapy designation by the FDA^93^. Several other examples of polypeptide fusion-inspired strategies exist, including the use of superhydrophilic zwitterionic peptides for enhancing circulation time and stability^94,95^. The use of polypeptides also offers an additional advantage over PEG owing to their inherent biodegradability. In other work, especially where intracellular delivery is desired, fusion to cell penetrating peptides has been explored as a viable strategy^26,96^. It should however be noted that immunogenicity can be a concern with certain peptide partners^23^.

The use of other fusion partners can endow structures with interesting properties related to targeting. For instance, Lee at al showed that proteins modified with tannic acid exhibit prolonged circulation time and appreciable accumulation in the heart^24^. Similarly, Zuchero et al. engineered Fc fragments as fusion partners that can bind the transferrin receptor and consequently transport proteins across the blood brain barrier^97^.

#### Unnatural amino acids (UAAs)

Incorporation of UAAs into protein structures is a particularly interesting area of work as they can be placed site-specifically, and their biorthogonal functional groups can be leveraged for chemical conjugation. For instance, this has allowed for controlled site-specific conjugation of PEG molecules^98,99^. In one case, Cho et al. incorporated an UAA with an acetyl functional group for reaction with oxime-functionalized PEG^100^. In their study, they screened a set of structures, each with a single UAA at one of 6 unique positions. A ~3-fold range in activity of the conjugated protein was observed depending on where the PEG was attached, showing the advantage of being able to control the position of PEG modification. In similarly inspired examples, the use of unnatural amino acids has enabled the site-specific installation of ADC payloads^40,101^, lipid groups^102^, and fusion proteins^103^. In another case, Sullivan et al. incorporated UAAs into proteins in a clustered fashion promoting enhanced and targeted cellular uptake of a therapeutic enzyme (yeast cytosine deaminase)^104^. Specifically, the group used the UAAs as chemical handles to conjugate multiple copies of epidermal growth factor receptor (EGFR)-targeting peptides to the protein surface in relatively close proximity to one another. This allowed for multivalent binding to the EGFR receptor, overexpressed on inflammatory breast cancer (IBC) cells, and ultimately enabled the delivery of the enzyme intracellularly. In a recent example, Wang et al. developed a strategy called proximity-enabled reactive therapeutics (PERx) for generating covalent protein drugs^105^. The authors incorporated a bioreactive amino acid fluorosulfate-L-tyrosine (FSY) into human programmed cell death protein-1 (PD-1) which could covalently attach to a proximal histidine on PD-L1 only upon PD-1-PD-L1 interaction. The resulting structures showed a dramatic increase in potency over the noncovalent wild-type PD-1^105^.

Challenges with UAAs are similar to those observed with site-specific mutagenesis, including the potential for mutations to decrease protein stability or activity. Moreover, the susceptibility of UAAs for inefficient incorporation often results in low protein yield^106^.

### Conclusions and outlook

Protein-based therapeutics have already revolutionized medicine and are increasingly growing in scope and impact. Each therapeutic has a set of desirable properties, including conformational and colloidal stability, sufficient circulation time, high potency, and lack of toxicity. The advent of chemical design strategies that enhance these characteristics has been indispensable to the field and has catalyzed its progress. In the future, we envision the continued use of established modifications, including PEGylation, ADCs, and fusions. We also envision the sustained growth of emerging strategies that improve certain properties without negatively impacting others. In this regard, further advances in computational capabilities that enable de novo therapeutic protein design will be instrumental in creating structures that exhibit desirable properties across the board. Considering that many modification strategies lead to heterogeneous end products, further research into site-specific conjugation remains highly necessary. These efforts are particularly important as each heterogeneous end product in a complex mixture can differ in its pharmacological property including safety and efficacy. We anticipate that intracellular therapeutic targets will also be an active area of research. This will put the spotlight on strategies that can mediate the delivery of proteins into cells, such as supercharging or modifying the surface with DNA. Once in the cell, the next hurdle to overcome will be attaining organelle-level specificity for therapeutic action. The further development of structures that can reach hard to target locations in the body, like the brain, will also unlock new capabilities in the field. The chemical design of orally bioavailable proteins will be a continued area of interest, helping to develop a more convenient delivery alternative to injections^107^. Taken together, these advances will fuel the sustained evolution of proteins in their role as essential tools in medicine.

## Supplementary information

Supplementary Information
