# Supplementary material for: Engineering protein-based therapeutics through structural and chemical design
Source: Nat Commun. 2023 Apr 27;14:2411. doi: 10.1038/s41467-023-38039-x (PMC10132957; doi:10.1038/s41467-023-38039-x)
Supplement: Supplementary file 1 — Supplementary Information [file 41467_2023_38039_MOESM1_ESM.pdf]

Engineering Protein-Based Therapeutics Through Structural and Chemical Design

Sasha B. Ebrahimi[1,*] and Devleena Samanta[2,*]

[1]Drug Product Development – Steriles, GlaxoSmithKline, Collegeville, PA 19426, USA

[2]Department of Chemistry, The University of Texas at Austin, Austin, TX 78712, USA

[*]Corresponding author email: sasha.b.ebrahimi@gsk.com, dsamanta@utexas.edu

Supplementary Table 1. Common FDA-approved variants of insulin 2

Supplementary Table 2. FDA-approved antibody-drug conjugates 3

Supplementary Table 3. Expanded list of protein-based therapeutics approved since 1982 4

All tables are created using information obtained from the PharmaCircle database.

Supplementary Table 1. Common FDA-approved variants of insulin

Therapeutic	Trade Name	Year of FDA Approval
insulin, human recombinant	Humulin	1982
insulin, human recombinant	Novolin	1991
insulin lispro	Humalog	1996
insulin, human recombinant	Velosulin	1999
insulin aspart	NovoLog	2000
insulin glargine	Lantus	2000
insulin glulisine	Apidra	2004
insulin detemir	Levemir	2005
insulin, human recombinant (inhalable)	Exubera	2006
insulin, human recombinant (inhalable)	Afrezza	2014
insulin degludec	Tresiba	2015
insulin glargine	Basaglar	2015
insulin glargine	Toujeo	2015
insulin aspart	Ryzodeg	2015
insulin aspart	Fiasp	2017
insulin lispro	Admelog	2017
insulin glargine	Lusduna	2017
insulin, human recombinant	Myxredlin	2019
insulin lispro	Lyumjev	2020
insulin glargine-yfgn	Semglee	2020
insulin glargine-aglr	Rezvoglar	2021

Supplementary Table 2. FDA-approved antibody-drug conjugates

**Therapeutic**	**Selected Trade Name**	**Selected Indications**	**Year of FDA Approval**
brentuximab vedotin	Adcetris	cancer, Hodgkin lymphoma	2011
trastuzumab emtansine	Kadcyla	breast cancer	2013
inotuzumab ozogamicin	Besponsa	acute lymphoblastic leukemia	2017
gemtuzumab ozogamicin	Mylotarg	cancer, acute myeloid leukemia	2017
moxetumomab pasudotox-tdfk	Lumoxiti	hairy cell leukemia	2018
fam-trastuzumab deruxtecan-nxki	Enhertu	breast cancer	2019
polatuzumab vedotin-piiq	Polivy	cancer, diffuse large B-cell lymphoma (DLBCL)	2019
enfortumab vedotin-ejfv	Padcev	cancer, renal	2019
sacituzumab govitecan-hziy	Trodelvy	breast cancer, triple-negative metastatic	2020
tisotumab vedotin	Tivdak	cervical cancer	2021
belantamab mafodotin	Blenrep	cancer, multiple myeloma	2021
loncastuximab tesirine-lpyl	Zynlonta	cancer, DLBCL	2021

Supplementary Table 3. Expanded list of protein-based therapeutics approved since 1982

Therapeutic	Selected Trade Names	Selected Indications	Year of FDA Approval
insulin, human recombinant	Humulin	diabetes	1982
somatrem	Protropin	growth hormone (GH) deficiency	1985
Interferon-α2b	Intron A	cancer, hairy cell leukemia	1986
interferon alfa 2a	Roferon	cancer, chronic myeloid leukemia	1986
digoxin immune Fab	Digibind	intoxication/ poisoning	1986
urofollitropin	Metrodin	infertility	1986
anti-inhibitor coagulant complex	Feiba	hemophilia A	1986
muromonab-CD3	Orthoclone OKT3	organ transplantation	1986
growth hormone, human recombinant (somatotropin)	Humatrope	GH deficiency	1987
alteplase	Activase	myocardial infarction	1987
interferon alfa-n3	Alferon N	genital warts	1989
epoetin alfa	Procrit	anemia	1989
anistreplase	Eminase	myocardial infarction	1989
interferon-γ1b (IFNγ)	Actimmune	chronic granulomatous disease, osteoporosis	1990
pegademase, bovine	Adagen	severe combined immunodeficiency	1990
filgrastim	Neupogen	neutropenia	1991
sargramostim	Leukine	bone marrow transplantation, stem cell mobilization	1991
onabotulinumtoxin A	Botox	hyperhidrosis	1991
antithrombin III, human	Thrombate III	antithrombin III deficiency	1991
alglucerase	Ceredase	Gaucher’s disease	1991
insulin, human recombinant	Novolin	diabetes	1991
octocog alfa	Recombinate	hemophilia A	1992
aldesleukin	Proleukin	cancer, renal cell carcinoma, metastatic	1992
interferon-β1b (rIFN-β)	Betaseron, Extavia	multiple sclerosis (MS)	1993
dornase alfa	Pulmozyme	cystic fibrosis/ respiratory tract infection	1993
octocog alfa	Kogenate	hemophilia A	1993
menotropin	Humegon	infertility	1994
pegaspargase	Oncaspar	acute lymphoblastic leukemia	1994
imiglucerase	Cerezyme	Gaucher’s disease	1994
abciximab	ReoPro	cardiac ischemia	1994
growth hormone, human recombinant (somatotropin)	Genotropin	GH deficiency	1995

growth hormone, human recombinant (somatotropin)	Norditropin	idiopathic short stature	1995
interferon-β1a (rIFN-β)	Avonex, Plegridy, Rebif	MS	1996
reteplase	Retavase	myocardial infarction	1996
growth hormone, human recombinant (somatotropin)	Saizen	GH deficiency	1996
growth hormone, human recombinant (somatotropin)	Serostim	cachexia, AIDS-related	1996
lipase<br>protease<br>amylase	Cotazym	exocrine pancreatic insufficiency	1996
Insulin lispro	Humalog	diabetes	1996
interferon alfacon-1	Infergen	hepatitis C	1997
follitropin alfa	Gonal	*in vitro* fertilization	1997
nonacog alfa	BeneFIX	hemophilia B	1997
oprelvekin	Neumega	thrombocytopenia	1997
becaplermin	Regranex	diabetic neuropathic ulcers	1997
rituximab	Rituxan	non-Hodgkin's lymphoma	1997
daclizumab beta	Zenapax	transplant rejection	1997
etanercept	Enbrel	juvenile arthritis	1998
lepirudin	Refludan	thrombocytopenia	1998
sacrosidase	Sucraid	enzyme deficiency (congenital sucrase-isomaltase deficiency)	1998
trastuzumab	Herceptin	breast cancer	1998
palivizumab	Synagis	infections, RSV	1998
basiliximab	Simulect	transplant rejection	1998
infliximab	Remicade	Crohn's disease	1998
poractant alfa	Curosurf	respiratory distress syndrome	1999
eptacog alfa	NovoSeven	hemophilia A	1999
growth hormone, human recombinant (somatotropin)	Nutropin	GH deficiency, adult	1999
denileukin diftitox	Ontak	cancer, CTCL cutaneous T-cell lymphoma	1999
moroctocog alfa	Refacto	hemophilia A	2000
choriogonadotropin alfa	Ovidrel	ovulation induction	2000
botulinum toxin type B	Myobloc	cervical dystonia	2000
octocog alfa	Helixate	hemophilia A	2000
tenecteplase	Tnkase	myocardial infarction	2000
insulin aspart	NovoLog	diabetes	2000
insulin glargine	Lantus	diabetes	2000
peginterferon alfa-2b	PEGIntron	hepatitis C	2001
darbepoetin alfa	Aranesp	anemia associated with renal failure	2001

anakinra	Kineret	inflammation/immune (deficiency of interleukin-1 receptor antagonist)	2001
drotrecogin alfa (activated)	Xigris	sepsis	2001
digoxin immune Fab	DigiFab	intoxication/poisoning	2001
alemtuzumab	Lemtrada (Originally called Campath)	MS	2001
interferon-β1a (rIFN-β)	Avonex, Plegridy, Rebif	MS	2002
pegylated interferon alfa-2a	Pegasys	hepatitis B, C	2002
rasburicase	Elitek	hyperuricemia	2002
pegfilgrastim	Neulasta	neutropenia	2002
dibotermin alfa	Infuse	fractures	2002
urofollitropin	Bravelle	infertility	2002
adalimumab	Humira	rheumatoid arthritis	2002
alefacept	Amevive	psoriasis	2003
growth hormone, human recombinant (somatotropin)	Zorbtive	short bowel syndrome	2003
collagen	CosmoDerm	skin wrinkles	2003
pegvisomant	Somavert	acromegaly	2003
octocog alfa	Advate	hemophilia A	2003
laronidase	Aldurazyme	Hurler syndrome	2003
agalsidase beta	Fabrazyme	Fabry disease	2003
efalizumab	Raptiva	psoriasis	2003
omalizumab	Xolair	asthma	2003
tositumomab and iodine 1131 tositumomab	Bexxar	non-Hodgkin’s lymphoma	2003
palifermin	Kepivance	oral mucositis	2004
lutropin alfa	Luveris	infertility	2004
lutropin alfa menotropin	Menopur	infertility	2004
follitropin beta	Follistim	infertility	2004
hyaluronidase, bovine testicular origin	Amphadase	adjuvant to increase the absorption and dispersion of other injected drugs	2004
hyaluronidase, ovine testicular origin	Vitrase	hypodermoclysis	2004
insulin glulisine	Apidra	diabetes	2004
natalizumab	Tysabri	MS, Crohn's disease	2004
cetuximab	Erbitux	colorectal cancer	2004
bevacizumab	Avastin	colorectal cancer	2004
mecasermin	Increlex	severe primary insulin-like growth factor deficiency	2005

abatacept	Orencia	rheumatoid arthritis	2005
mecasermin rinfabate	Iplex	GH deficiency (growth hormone)	2005
galsulfase	Naglazyme	Maroteaux-Lamy syndrome	2005
hyaluronidase, human recombinant	Hylenex	adjuvant agent to enhance drug delivery	2005
hyaluronidase, bovine testicular origin	Hydase	adjuvant to increase the absorption and dispersion of other injected drugs	2005
insulin detemir	Levemir	diabetes	2005
alglucosidase alfa	Myozyme	Pompe disease	2006
growth hormone, human recombinant (somatotropin)	Omnitrope	GH deficiency	2006
idursulfase	Elaprase	Hunter syndrome	2006
insulin, human recombinant	Exubera Inhaled Insulin	diabetes	2006
ranibizumab	Lucentis	age-related macular degeneration (AMD), Wet	2006
panitumumab	Vectibix	colorectal cancer	2006
thyrotropin alfa	Thyrogen	thyroid cancer	2007
growth hormone, human recombinant (somatotropin)	Valtropin	GH deficiency	2007
peg-erythropoietin beta	Mircera	anemia, renal failure	2007
eculizumab	Soliris	paroxysmal nocturnal hemoglobinuria	2007
moroctocog alfa	Xyntha	hemophilia A	2008
growth hormone, human recombinant (somatotropin)	Accretropin	Turner syndrome	2008
rilonacept	Arcalyst	Muckle-Wells syndrome	2008
romiplostim	Nplate	idiopathic thrombocytopenic purpura	2008
C1-inhibitor, human plasma-derived (C1 esterase inhibitor)	Cinryze	angioedema, hereditary prophylaxis	2008
factor IIa, human recombinant (thrombin)	Recothrom	hemostasis	2008
certolizumab pegol	Cimzia	Crohn's disease	2008
Interferon-β1b (rIFN-β)	Betaseron, Extavia	MS	2009
antithrombin alfa	ATryn	antithrombin III deficiency	2009
ecallantide	Kalbitor	angioedema, hereditary	2009
abobotulinumtoxin A	Dysport	skin wrinkles	2009
incobotulinumtoxinA	Xeomin	sialorrhea	2009
C1-inhibitor, human plasma-derived (C1 esterase inhibitor)	Berinert	angioedema	2009

lipase<br>protease<br>amylase	Creon	exocrine pancreatic insufficiency	2009
lipase<br>protease<br>amylase	Zenpep	exocrine pancreatic insufficiency	2009
ustekinumab	Stelara	Crohn's disease	2009
ofatumumab	Arzerra	cancer, chronic lymphocytic leukemia (CLL)	2009
canakinumab	Ilaris	cryopyrin-associated periodic syndromes	2009
golimumab	Simponi	psoriatic arthritis	2009
velaglucerase alfa	VPRIV	Gaucher's disease	2010
alglucosidase alfa	Lumizyme	Pompe disease	2010
lipase<br>protease<br>amylase	Pancreaze	exocrine pancreatic insufficiency	2010
pegloticase	Krystexxa	gout	2010
collagenase AUX-I<br>collagenase AUX-II	Xiaflex	dupuytren's disease	2010
tocilizumab	Actemra	rheumatoid arthritis	2010
denosumab	Prolia	osteoporosis	2010
denosumab	Xgeva	bone diseases	2010
belatacept	Nulojix	transplant rejection	2011
aflibercept	Eylea	diabetic retinopathy	2011
factor XIII, human	Corifact	coagulopathy	2011
asparaginase Erwinia chrysanthemi	Erwinaze	acute lymphoblastic leukemia	2011
belimumab	Benlysta	systemic lupus erythematosus	2011
ipilimumab	Yervoy	Melanoma	2011
brentuximab vedotin	Adcetris	cancer, anaplastic large cell lymphoma	2011
tbo-filgrastim	Granix	neutropenia	2012
taliglucerase alfa	Elelyso	Gaucher's disease	2012
ocriplasmin	Jetrea	vitreomacular adhesion	2012
aflibercept	Zaltrap	cancer, colorectal, metastatic	2012
glucarpidase	Voraxaze	chemo side effects (methotrexate overdosage)	2012
lipase<br>protease<br>amylase	Viokace	exocrine pancreatic insufficiency	2012
lipase<br>protease<br>amylase	Ultresa	exocrine pancreatic insufficiency	2012
lipase<br>protease<br>amylase	Pertzye	exocrine pancreatic insufficiency	2012
pertuzumab	Perjeta	breast cancer	2012
raxibacumab	Raxibacumab	anthrax	2012

nonacog gamma	Rixubis	hemophilia B	2013
turoctocog alfa	NovoEight	hemophilia A	2013
catridecacog	Tretten	thrombosis	2013
obinutuzumab	Gazyva	cancer, CLL	2013
trastuzumab emtansine	Kadcyla	breast cancer	2013
interferon-β1a (rIFN-β)	Avonex, Plegridy, Rebif	MS	2014
elosulfase alfa	Vimizim	Morquio syndrome	2014
efmoroctocog alfa	Eloctate	hemophilia A	2014
susoctocog alfa	Obizur	hemophilia A	2014
conestat alfa	Ruconest	angioedema	2014
eftrenonacog alfa	Alprolix	hemophilia B	2014
metreleptin	Myalept	lipodystrophy	2014
albiglutide	Tanzeum	diabetes	2014
dulaglutide	Trulicity	diabetes	2014
insulin, human recombinant	Afrezza Inhaled Insulin	diabetes	2014
ramucirumab	Cyramza	stomach cancer	2014
vedolizumab	Entyvio	Crohn's disease	2014
blinatumomab	Blincyto	acute lymphoblastic leukemia	2014
nivolumab	Opdivo	melanoma	2014
pembrolizumab	Keytruda	cancer, non-small cell lung cancer (NSCLC), metastatic	2014
siltuximab	Sylvant	Castleman's disease	2014
asfotase alfa	Strensiq	hypophosphatasia	2015
filgrastim-sndz	Zarxio	neutropenia	2015
sebelipase alfa	Kanuma	lysosomal acid lipase deficiency	2015
trenonacog alfa	Ixinity	hemophilia B	2015
rurioctocog alfa pegol	Adynovate	hemophilia A	2015
simoctocog alfa	Nuwiq	hemophilia A	2015
growth hormone, human recombinant (somatotropin)	Zomacton	GH deficiency, child	2015
parathyroid hormone, human recombinant	Natpara	hypocalcemia	2015
lipase	RELiZORB	exocrine pancreatic insufficiency	2015
insulin degludec	Tresiba	diabetes	2015
insulin glargine	Basaglar	diabetes	2015
insulin glargine	Toujeo	diabetes	2015
insulin aspart	Ryzodeg	diabetes	2015
necitumumab	Portrazza	cancer, NSCLC	2015
mepolizumab	Nucala	asthma	2015
evolocumab	Repatha	hyperlipidemia	2015
elotuzumab	Empliciti	multiple myeloma	2015
daratumumab	Darzalex	cancer, multiple myeloma	2015
alirocumab	Praluent	high cholesterol, familial heterozygous	2015
idarucizumab	Praxbind	coagulopathy	2015
secukinumab	Cosentyx	psoriasis	2015

dinutuximab	Unituxin	neuroblastoma	2015
lonoctocog alfa	Afstyla	hemophilia A	2016
albutrepenonacog alfa	Idelvion	hemophilia B	2016
etanercept-szzs	Erelzi	arthritis	2016
octocog alfa	Kovaltry	hemophilia A	2016
insulin glargine and lixisenatide	Soliqua	diabetes	2016
liraglutide	Xultophy	diabetes	2016
adalimumab-atto	Amjevita	rheumatoid arthritis	2016
bezlotoxumab	Zinplava	infections, *Clostridium difficile*	2016
ixekizumab	Taltz	psoriasis	2016
atezolizumab	Tecentriq	cancer, NSCLC	2016
infliximab-dyyb	Inflectra	Crohn's disease	2016
daclizumab beta	Zinbryta	MS	2016
reslizumab	Cinqair	asthma	2016
olaratumab	Lartruvo	cancer, soft tissue sarcoma	2016
obiltoxaximab	Anthim	infections, anthrax	2016
cerliponase alfa	Brineura	Batten disease	2017
vestronidase alfa-vjbk	Mepsevii	mucopolysaccharidosis	2017
nonacog beta pegol	Rebinyn	hemophilia B	2017
thrombin, human chondroitin sulfate collagen	Hemoblast	hemostasis	2017
keratin, human derived	KeraStat	radiation side effects<br>wound care	2017
C1-inhibitor, human plasma-derived (C1 esterase inhibitor)	Haegarda	angioedema	2017
insulin aspart	Fiasp	diabetes	2017
Insulin lispro	Admelog	diabetes	2017
bevacizumab-awwb	Mvasi	colorectal cancer	2017
trastuzumab-dkst	Ogivri	breast cancer	2017
adalimumab-adbm	Cyltezo	rheumatoid arthritis	2017
ocrelizumab	Ocrevus	MS, relapsing remitting	2017
guselkumab	Tremfya	psoriasis	2017
benralizumab	Fasenra	asthma	2017
dupilumab	Dupixent	asthma	2017
durvalumab	Imfinzi	cancer, NSCLC	2017
sarilumab	Kevzara	rheumatoid arthritis	2017
avelumab	Bavencio	cancer, Merkel cell carcinoma, metastatic	2017
emicizumab-kxwh	Hemlibra	hemophilia A	2017
rituximab	Rituxan Hycela	follicular lymphoma	2017
infliximab-abda	Renflexis	follicular lymphoma	2017
Infliximab-qbtx	Ixifi	Crohn's disease	2017
brodalumab	Siliq	psoriasis	2017
inotuzumab ozogamicin	Besponsa	acute lymphoblastic leukemia	2017

gemtuzumab ozogamicin	Mylotarg	cancer, acute myeloid leukemia	2017
insulin glargine	Lusduna	diabetes	2017
filgrastim-aafi	Nivestym	neutropenia	2018
pegfilgrastim-cbqv	Udenyca	neutropenia	2018
pegfilgrastim-jmdb	Fulphila	neutropenia	2018
andexanet alfa	Andexxa	anticoagulation reversal	2018
vonicog alfa	Vonvendi	von Willebrand disease	2018
epoetin alfa-epbx	Retacrit	snemia	2018
cenegermin-bkbj	Oxervate	eye diseases, corneal (neurotrophic keratitis)	2018
tagraxofusp-erzs	Elzonris	cancer, BPDCN (blastic plasmacytoid dendritic cell neoplasm)	2018
calaspargase pegol-mknl	Asparlas	acute lymphoblastic leukemia	2018
pegvaliase-pqpz	Palynziq	phenylketonuria	2018
elapegademase-lvlr	Revcovi	SCID (severe combined immunodeficiency)	2018
trastuzumab-pkrb	Herzuma	breast cancer	2018
adalimumab-adaz	Hyrimoz	rheumatoid arthritis	2018
galcanezumab-gnlm	Emgality	migraine	2018
cemiplimab-rwlc	Libtayo	cancer, basal cell metastatic	2018
ravulizumab-cwvz	Ultomiris	paroxysmal nocturnal hemoglobinuria	2018
rituximab-abbs	Truxima	non-Hodgkin’s lymphoma	2018
ibalizumab-uiyk	Trogarzo	infections, HIV/AIDS	2018
fremanezumab-vfrm	Ajovy	migraine	2018
lanadelumab-flyo	Takhzyro	angioadema	2018
tildrakizumab-asmn	Ilumya	psoriasis	2018
erenumab-aooe	Aimovig	migraine	2018
mogamulizumab-kpkc	Poteligeo	mycosis fungoides	2018
burosumab-twza	Crysvita	X-linked hypophosphatemia	2018
emapalumab-lzsg	Gamifant	genetic disorders (hemophagocytic lymphohistiocytosis)	2018
moxetumomab pasudotox-tdfk	Lumoxiti	hairy cell leukemia	2018
pegfilgrastim-bmez	Ziextenzo	neutropenia	2019
turoctocog alfa pegol	Esperoct	hemophilia A	2019
etanercept-ykro	Eticovo	arthritis	2019
luspatercept-aamt	Reblozyl	anemia	2019
prabotulinumtoxinA-xvfs	Jeuveau	skin wrinkles	2019
insulin, human recombinant	Myxredlin	diabetes	2019
bevacizumab-bvzr	Zirabev	colorectal cancer, glioblastoma multiforme, heptacellular carcinoma	2019

trastuzumab-anns	Kanjinti	breast cancer	2019
trastuzumab-dttb	Ontruzant	breast cancer	2019
trastuzumab-qyyp	Trazimera	breast cancer	2019
adalimumab-afzb	Abrilada	rheumatoid arthritis	2019
adalimumab-bwwd	Hadlima	rheumatoid arthritis	2019
adalimumab-afzb	Abrilada	rheumatoid arthritis	2019
caplacizumab-yhdp	Cablivi	thrombotic thrombocytopenic purpura	2019
risankizumab-rzaa	Skyrizi	psoriasis	2019
brolucizumab-dbll	Beovu	wet macular degeneration	2019
rituximab-pvvr	Ruxience	non-Hodgkin's Lymphoma	2019
infliximab-axxq	Avsola	rheumatoid arthritis	2019
romosozumab-aqqg	Evenity	osteoporosis	2019
crizanlizumab-tmca	Adakveo	prevention of vaso-occlusive crises (VOCs) in patients with sickle cell disease	2019
fam-trastuzumab deruxtecan-nxki	Enhertu	breast cancer	2019
polatuzumab vedotin-piiq	Polivy	cancer, DLBCL diffuse large B-cell lymphoma	2019
enfortumab vedotin-ejfv	Padcev	cancer, renal transitional cell carcinoma	2019
pegfilgrastim-apgf	Nyvepria	neutropenia	2020
activated eptacog beta	Sevenfact	hemophilia A	2020
somapacitan-beco	Sogroya	GH deficiency, adult (growth hormone deficiency)	2020
peanut arachis hypogaea Allergen-dnfp	Palforzia	peanut allergy	2020
collagenase clostridium histolyticum-aaes	Qwo	cellulite	2020
insulin lispro	Lyumjev	diabetes	2020
insulin glargine-yfgn	Semglee	diabetes	2020
trastuzumab	Phesgo	breast cancer	2020
adalimumab-fkjp	Hulio	rheumatoid arthritis	2020
satralizumab	Enspryng	neuromyelitis optica	2020
ofatumumab	Kesimpta	MS, relapsing remitting	2020
tafasitamab	Monjuvi	cancer, DLBCL diffuse large B-cell lymphoma	2020
naxitamab-gqgk	Danyelza	neuroblastoma	2020
eptinezumab-jjmr	Vyepti	migraine	2020
rituximab-arrx	Riabni	non-Hodgkin's lymphoma	2020
teprotumumab-trbw	Tepezza	thyroid eye disease	2020
ansuvimab-zykl	Ebanga	infections, Ebola	2020
inebilizumab-cdon	Uplizna	neuromyelitis optica	2020
margetuximab-cmkb	Margenza	breast cancer	2020
isatuximab-irfc	Sarclisa	cancer, multiple myeloma	2020

atoltivimab odesivimab-ebgn maftivimab	Inmazeb	infections, Ebola	2020
sacituzumab govitecan-hziy	Trodelvy	cancer, breast, triple-negative, metastatic	2020
ropeginterferon alfa-2b	Besremi	polycythemia vera	2021
efgartigimod alfa	Vyvgart	myasthenia gravis	2021
avalglucosidase alfa	Nexviazyme	Pompe disease	2021
lonapegsomatropin-tcgd	Skytrofa	GH deficiency	2021
asparaginase erwinia chrysanthemi-rywn	Rylaze	acute lymphoblastic leukemia	2021
insulin glargine-aglr	Rezvoglar	diabetes	2021
adalimumab-aqvh	Yusimry	rheumatoid arthritis	2021
ranibizumab	Susvimo	AMD, wet	2021
ranibizumab-nuna	Byooviz	macular degeneration	2021
anifrolumab	Saphnelo	systemic lupus erythematosus	2021
tralokinumab	Adbry	atopic dermatitis	2021
evinacumab-dgnb	Evkeeza	high cholesterol	2021
tezepelumab-ekko	Tezspire	asthma	2021
dostarlimab	Jemperli	cancer, solid	2021
amivantamab-vmjw	Rybrevant	cancer, NSCLC	2021
aducanumab-avwa	Aduhelm	Alzheimer's disease	2021
tisotumab vedotin	Tivdak	cervical cancer	2021
belantamab mafodotin	Blenrep	cancer, multiple myeloma	2021
loncastuximab tesirine-lpyl	Zynlonta	cancer, DLBCL	2021
filgrastim-ayow	Releuko	neutropenia	2022
tebentafusp-tebn	Kimmtrak	cancer, uveal melanoma metastatic	2022
bevacizumab-maly	Alymsys	colorectal cancer	2022
nivolumab/ relatlimab-rmbw	Opdualag	melanoma	2022
sutimlimab-jome	Enjaymo	cold agglutinin disease	2022
faricimab-svoa	Vabysmo	diabetic macular edema	2022
